# Agent-Based Models of Strategies for the Emergence and Evolution of Grammatical Agreement

**DOI:** 10.1371/journal.pone.0058960

**Published:** 2013-03-18

**Authors:** Katrien Beuls, Luc Steels

**Affiliations:** 1 Artificial Intelligence Laboratory, Vrije Universiteit Brussel, Brussels, Belgium; 2 ICREA - Institut de Biologia Evolutiva (UPF-CSIC), Barcelona, Spain; 3 Sony Computer Science Laboratory, Paris, France; Universitat Pompeu Fabra, Spain

## Abstract

Grammatical agreement means that features associated with one linguistic unit (for example number or gender) become associated with another unit and then possibly overtly expressed, typically with morphological markers. It is one of the key mechanisms used in many languages to show that certain linguistic units within an utterance grammatically depend on each other. Agreement systems are puzzling because they can be highly complex in terms of what features they use and how they are expressed. Moreover, agreement systems have undergone considerable change in the historical evolution of languages. This article presents language game models with populations of agents in order to find out for what reasons and by what cultural processes and cognitive strategies agreement systems arise. It demonstrates that agreement systems are motivated by the need to minimize combinatorial search and semantic ambiguity, and it shows, for the first time, that once a population of agents adopts a strategy to invent, acquire and coordinate meaningful markers through social learning, linguistic self-organization leads to the spontaneous emergence and cultural transmission of an agreement system. The article also demonstrates how attested grammaticalization phenomena, such as phonetic reduction and conventionalized use of agreement markers, happens as a side effect of additional economizing principles, in particular minimization of articulatory effort and reduction of the marker inventory. More generally, the article illustrates a novel approach for studying how key features of human languages might emerge.

## Introduction

Human languages use a variety of syntactic means to convey meaning beyond that covered by individal words. The best studied example concerns sequential ordering. For example, in the phrase *a beautiful girl*, the article, adjective and noun are adjacent to each other and this conveys that they belong to the same noun phrase. But there are other syntactic mechanisms that are just as common, in particular grammatical agreement [1], [2]. For example, in the French phrase *une belle fille* (a beautiful girl), the gender of the noun *fille* (girl) is feminine and singular, and these features reappear with the adjective *belle* (beautiful-FEM-SG), and the article *une* (a-FEM-SG). Languages with strong agreement systems, such as Latin, can have a freer word order because they rely less on sequential ordering to communicate syntactic structure.

The variation and complexity of human grammatical agreement systems is fascinating [2], [3]. Some languages (such as Japanese) use virtually no agreement whereas others (such as Latin) use it abundantly. The features used by agreement systems also vary across languages. Features such as person, gender, number, animacy, definiteness, case and countability, are typical for Indo-European languages but other languages, such as Bantu languages [4], use entirely different ones. A famous example is the category of ‘women, water, fire, violence, and exceptional animals’, which is used in the classifier and agreement system of Dyirbal (an Australian aboriginal language) [5]. The linguistic forms that are used to overtly mark agreement features vary considerably as well, and choices may take different aspects of the syntactic context into account [6]. Forms also undergo change, even up to a point where a complex agreement system may erode, as happened in the transition from Old to Middle English [7].

Here we are interested in the question how and why such agreement systems can originate and we thus address one of the key puzzles in the cultural evolution of grammatical language. We do not want to perform a historical reconstruction of stages in the evolution of agreement systems, which has been carried out by many researchers already [8], [9], but to construct theoretical models of the cognitive strategies and cultural processes that are sufficient to see the key characteristics of grammatical agreement arise. The models take the form of a population of agents which play language games about objects perceived in their environment and they have been operationalized and tested in computer simulations. Agent-based models are used with increasing success to study issues in the origins of language [10]–[12]. They are complementary to population models based on aggregate quantities, which have recently also become prominent [13]. In the present experiments, agents are provided with a vocabulary but no agreement system nor any other kind of grammar. They are also provided with cognitive operations needed for concept formation and the invention and learning of linguistic forms. A strategy using these operations is considered to be an adequate model to explain the origins of grammatical agreement if the characteristic properties of agreement systems arise in the shared artificial languages that the agents construct while playing language games.

What are the characteristic properties of agreement systems that we should target? Obviously we should see a system of markers emerge and we want to understand how the semantic features expressed by these markers become shared in a population. But we are also interested to model phenomena systematically observed in the evolution of grammatical agreement systems in natural languages. Historical linguists have shown that agreement markers invariably derive from reusing existing words, such as pronouns or classifiers [14], [15]. They then undergo two types of evolutionary processes: (i) The markers derived from independent words become shorter, they loose part of their form, then become clitics and later affixes [16], [17]. For example, the Diyrbal (Australian aboriginal) agreement marker *m-* evolved from the classifier *mayi*, which means ‘non-flesh food’ [18]. (ii) The features that agreement markers express, which are initially semantically grounded, invariably become more abstract and get to be used in a purely conventional manner [19]. For example, the masculine/feminine gender distinction has its basis in male/female sex, but is then arbitrarily applied to inanimate objects, so that table might be masculine in one language (German: *der Tisch*) but feminine in another (French: *la table*).

The present article contributes to the debate whether the structure of language is a consequence of its function and usage in communication, operating within the processing constraints of human embodiment and cognition, or whether function, processing, collective dynamics, and performance are irrelevant to explain universal features of language, such as agreement marking. The latter position is common in generative syntax [20]. Here we defend the first position and argue that agreement systems play a crucial role in damping combinatorial complexity in parsing and semantic ambiguity in interpretation. Grammaticalization processes lead to economization of an agreement system, not because individual language users consciously optimize their language but because the variants due to phonological reduction and re-analysis undergo positive cultural selection when they lead to a minimization of articulatory and auditory effort or a reduction of the marker inventory.

The rest of this introduction first presents a language game to investigate the role of agreement systems and proposes a hypothesis about the function of agreement systems.

### The Language Game

All computational models presented here involve a population of agents 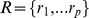 with p = 10. Two agents are randomly chosen to play the role of speaker and hearer respectively. Games are played within the context of a particular situation. A situation consists of a set of objects with various properties and agents are assumed to be able to construct a situation model based on perception, pattern recognition, object detection and low level feature analysis and categorisation, as in [21], [22]. The situation model represents the objects in the context as well as their properties using standard predicate calculus (see methods section for details). For example, the object 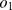 could have the properties 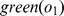, 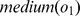, and 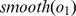. The properties reflect values of attributes, such as 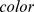, 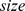, or 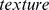. Each object is an instance of a certain type, and a type defines the possible attributes and values of its instances.

Agents are initialized with a shared pre-defined vocabulary consisting of associations between a set of properties, and a word, which consists of a random string of characters. For example, *toubuta* could mean 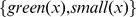, where x is a variable to be bound to an object in the situation model. Pre-defining a shared vocabulary is justified because many other agent-based models of vocabulary formation now exist [23] and it allows us to focus immediately on the issue of the emergence of an agreement system. The vocabulary (and later on the grammar) is implemented using Fluid Construction Grammar [12] but details of the implementation are not important to understand the general argument of the paper (see supplementary information in Link S1).

**Figure 1:**
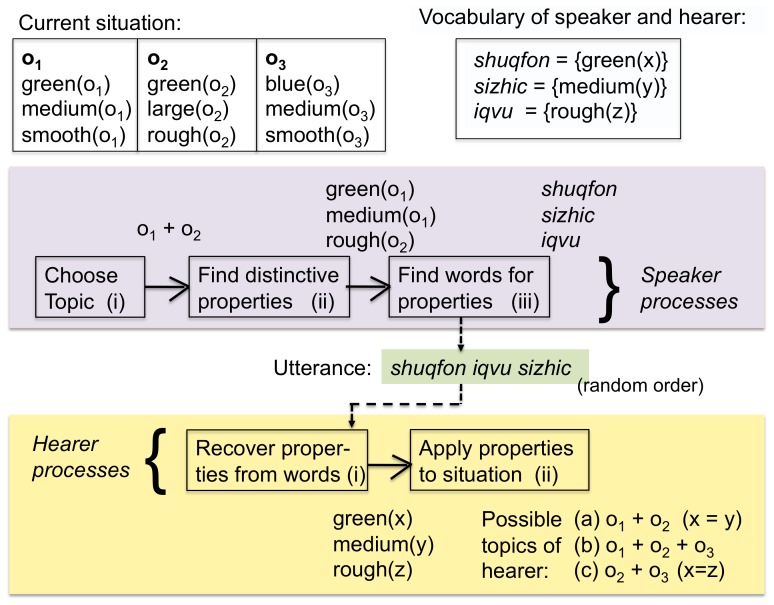
The language game. The situation contains three objects: 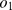 which has the properties 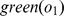, 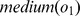, and 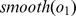, 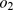 with properties 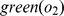, 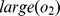, and 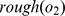 and 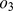 with 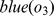, 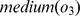, and 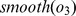. The speaker has chosen 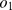 and 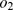 as topic and expresses the set of distinctive properties 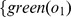, 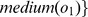 to identify 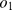 and 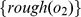 to identify 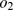. After vocabulary lookup the speaker finds that the word *shuqfon* covers 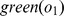, *sizhic* covers 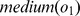 and *iqvu* covers 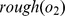. The utterance is therefore *shuqfon iqvu sizhic*. The hearer looks up these words in his own vocabulary and recovers 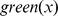, 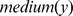, 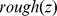, where 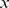, 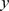 and 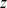 are variables to be bound to objects in the situation model. In the current situation model, the hearer finds that the topic can be either (a) 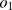 and 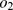 (implying that 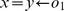 and 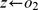) or (b) 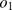, 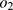, and 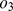 (so that 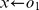, 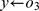, 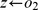), or (c) 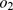 and 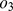 (so that 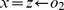 and 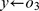)

Agents play a game of reference, also known as the Naming Game, which has been used in many earlier investigations of language dynamics [24], [25]. The main novelty of the present game is that the topic can consist of more than one object. The steps in the game are as follows (see Figure 1):

- The *speaker* selects a subset of the objects in the current situation to act as the topic of the utterance. (ii) The speaker looks for a distinctive combination of properties for each of the objects which are part of the topic. A distinctive combination is a set of properties which are true for that object but not for any other object in the current situation. (iii) The speaker retrieves the minimal set of words in the vocabulary that covers the chosen properties, which implies that words with the largest coverage are preferred. A word can possibly cover more properties than those that are distinctive, but in that case these other properties must also be true of the object the word refers to. The speaker then utters these words in a random order. Although there is unavoidably a sequential ordering to the words, this does not carry any meaning, i.e. agents use a word-order free language.

- Next, the agent chosen as *hearer* goes through the following steps: (i) The hearer looks up the words in his own vocabulary and thus reconstructs what properties have been communicated by the speaker. (ii) The hearer identifies which objects in the present situation satisfy these properties by matching the reconstructed meaning with the facts stored in his situation model, and points to these objects. The game is a success if the objects pointed to by the hearer are those initially chosen by the speaker. The game fails if this is not the case or if the utterance remains semantically ambiguous, i.e. if there is more than one possible interpretation that fits with the current situation model.

### What agreement is for

**Figure 2:**
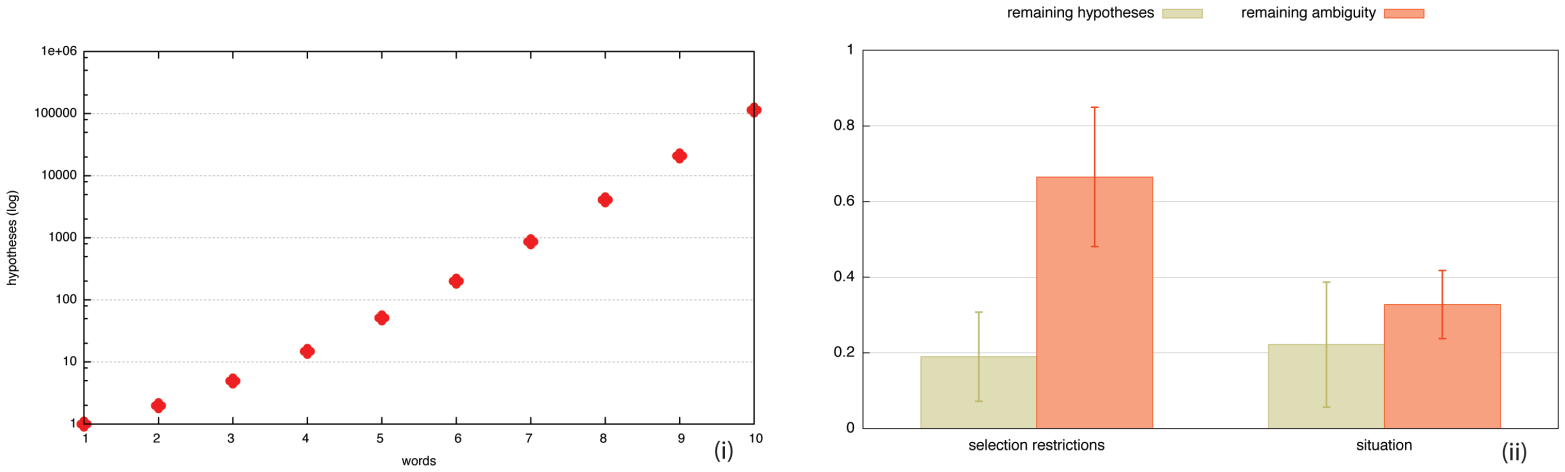
Combinatorial complexity of interpretation. *Left*: The number of possible hypotheses grows exponentially with the number of words as predicted by equation (1). *Right*: Percentage of remaining hypotheses and remaining semantic ambiguity after the application of selection restrictions (left) or the consultation of the situation model (right), for 50 game series with 10 agents and 5000 games in total (1000 per agent). Average values are shown with standard deviations. Both sources of information reduce the set of possible hypotheses in the search space but significant semantic ambiguity remains.

Although the language game being used here looks deceptively simple, there is the potential for a combinatorial explosion and semantic ambiguity. The hearer does not know how many objects the speaker is talking about and the utterance does not communicate which words are about the same object. Hence, all possible combinations must be tried by the hearer to find those that fit with the current situation. The number of combinations 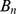 is equal to the number of partitions of the set D of words in an utterance of size n, where a partition of D is defined as a set of nonempty, pairwise disjoint subsets of D whose union is D. 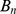 is known as the Bell number and defined using the following equation [26]:
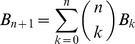
(1)with 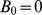 and 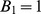. 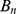 grows exponentially with the number of words (see Figure 2). It means that the sentence you are now reading (which contains 20 words) generates 51,724,158,235,372 partitions and hence possible interpretations. Agents could refrain from using multiple words and code all properties of an object with single ‘holistic’ words, however this would increase the size of the vocabulary considerably because all possible combinations of meanings then need a separate word, and it would not allow them to deal with an open set of objects or with novel combinations of distinctive properties. So combinatorial complexity is the price to pay for a compositional language.

Of course, human language users exploit semantic knowledge, i.e. constraints which are generally applicable, independently of the context. For example, in the utterance *brown good book idea*, we know that *brown* and *book* most likely belong together because it makes sense for a book to be brown whereas ideas are colorless. The dependency of *good* remains ambiguous because both an idea and a book can be good. Human language users also exploit the situation to make sense of an utterance. If we see a brown book before us, then it is obvious that *brown* and *book* are co-referential in the present context.

We have investigated the effectiveness of both strategies for the present language game. The capacity to apply selection restrictions has been operationalized by giving agents access to an ontology defining the possible types of objects and their possible attributes and values. Because each type has only a limited set of properties and objects are constructed on the basis of types, the agent can eliminate all interpretations which contain properties that do not belong to the same type. The capacity to apply constraints coming from the situation model is already a necessary step in the interpretation process (step iii), so agents are capable to use that for pruning hypotheses as well.

However, despite applying selection restrictions and using the situation model for semantic disambiguation, combinatorial complexity and remaining ambiguity remains significant in the present model (see Figure 2). Indeed this is the case for most human language utterances as well. Moreover, in displaced communication, i.e. communication when speaker and hearer do not share the same context, the hearer cannot build a grounded model of the current situation and hence there is no way to eliminate hypotheses or ambiguity using the context. The only way to deal reliably with combinatorial complexity and semantic ambiguity is therefore through some form of grammar and we hypothesize that agreement systems have developed as one of the ways in which natural languages do this. Other strategies are used as well, for example, exploiting sequential ordering, stress, or intonation patterns. Agreement systems are therefore not absolutely necessary, just a possibility, and most human languages use a combination of different strategies.

## Experiments

We now present a series of experiments exploring strategies by which agreement systems may emerge and culturally propagate. It is known from historical data that agreement systems initially arise by reusing existing words as markers [14], [15]. To understand the underlying mechanisms, we have done a first series of experiments in order to model this process. As a first step we investigate formal markers, i.e. markers without any meaning, in order to establish the basic dynamics and cognitive mechanisms underlying agreement. Then we look at meaningful markers. First, markers invented *de novo* and next markers that arise by the recruitment of existing words. But the story does not end here. It has been universally observed that agreement markers evolve culturally in two ways: The form of the markers shrinks, minimizing the effort in speech articulation, and their meaning becomes more general and conventional. We have therefore done a second series of experiments modeling these grammaticalization processes. One experiment focuses on phonological reduction and a second one on coercion.

### The Sticker Principle

Attaching stickers to objects is a straightforward common sense idea for organizing them. For example, students that share a kitchen in a student house could paste a colored sticker on those food items in the refrigerator that they want to signal as their own. We can view the markers used in grammatical agreement as stickers. For example, the Swahili phrase *ki-kapu ki-kubwa ki-moja* (ki.SG-basket ki-large ki-one) [4] has a marker *ki* added to all words in which the same object, namely the basket, is implicated. Another example is the Latin *ill-arum du-arum bon-arum femin-arum* (those-arum two-arum good-arum women-arum), where the marker *-arum* is attached to all words which introduce properties of the same referent.

**Figure 3:**
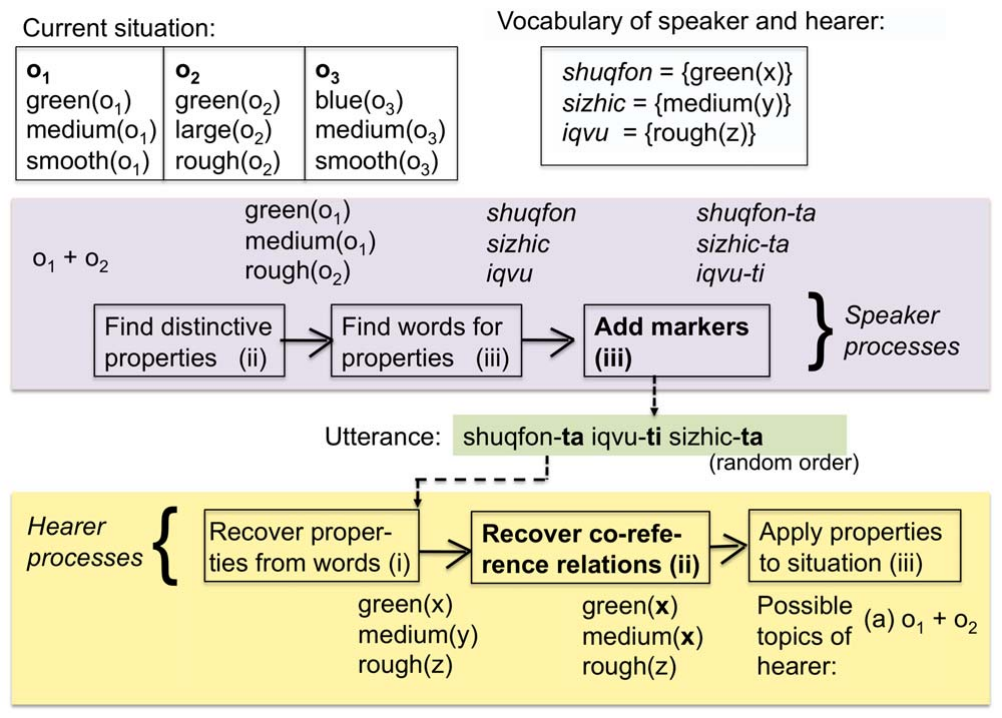
Formal marker strategy. The production of an utterance by the speaker now involves an extra step (step iii) to identify words that are about the same object and add markers to them. In parsing, the hearer uses the markers to reconstruct the co-reference relations, and makes the variables for properties refering to the same object equal. In the present example, y and x are co-referential because the words that introduce properties with these variables contain the same markers. Only one possible interpretation, namely that the topic is 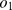 and 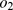 then remains.

We have operationalized the sticker principle in terms of a *formal marker strategy* (see Figure 3 and supplementary information in Link S1), in which agents go through the following steps:

- The speaker detects the need for a marker by re-entering the utterance he is about to say in his own parsing and interpretation system, in order to simulate the difficulties the hearer might encounter. When the speaker notices combinatorial complexity or semantic ambiguity, he invents a random string to act as a marker (for example *-waeyyaes* or *-riizu*) and builds a new grammatical construction. The construction adds a marker after each word that introduces properties about the same object. Once the speaker has created a marker construction, he applies it to the current utterance. The speaker also adds the new construction to his inventory. In subsequent games, constructions are applied routinely, even in situations where markers are not strictly needed, thus avoiding the costly computation needed for simulating the hearer's potential difficulties.
- The hearer automatically applies the markers in his own inventory when they occur in an utterance and this automatically prunes possible hypotheses so that combinatorial complexity and semantic ambiguity are avoided. When the hearer encounters an unknown string in the utterance, he checks whether this string occurs more than once and this is taken as a sign that the unknown string is an agreement marker. So the hearer can use it straight away to prune possible hypotheses. He also constructs a new grammatical construction for this new marker and from then on uses it routinely as well, not only in parsing but also in his own production.

This strategy can be expected to lead to the invention of new markers and their propagation in the population. However, an important issue is left unresolved. If all agents invent their own, there are unavoidably going to be many markers circulating in the language because agents have only local interactions and hence no global overview of the markers already invented by other agents. Consequently, the marker inventory grows with the size of the population and language learners need more and more time to learn all the markers that are in use [25]. Moreover the routine application of markers will slow down because so many of them need to be stored. In contrast, human languages have only very few agreement markers and they are shared by all speakers of the language.

How can a distributed population of agents agree on a shared set of markers without central control nor prior design? This problem is an instance of the general problem of convention sharing which has already been studied intensively using agent-based models. Various solutions are known, including the use of frequency [27], voter models [28], lateral inhibition [23], [29], shaping [30] and cross-situational learning [31]. The solution adopted here is based on lateral inhibition. Agents maintain a score 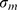 between 0.0 and 1.0 for every marker 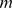. When choosing which marker to use, the speaker prefers the marker that has the highest score and that was not yet used in the same utterance. When two markers have the same highest score, a random choice is made. The initial score for a newly introduced association is 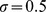. The hearer (but not the speaker) increments this score whenever a marker 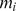 appears in an utterance and decreases the score of all other non-used markers 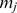 using the following equations with alignment rate 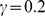:
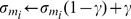
(2)

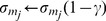
(3)


**Figure 4:**
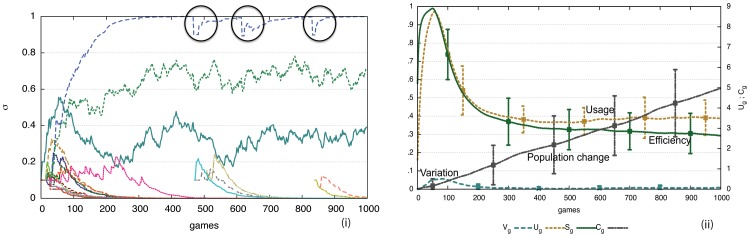
Performance of the formal marker strategy. i) Computer simulations for 50 game series involving 10 agents playing 1000 language games (200 per agent). Average values and standard deviations are shown. At each time point only two agents play a game, although the model works just as well with parallel interactions. Usage inventory 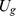 reaches a peak after 50 games (10 per agent) after which it gets damped to an optimum of three markers (because the maximum number of objects chosen as topic is 3) due to the lateral inhibition dynamics, before increasing slightly when new agents come into the population. Variation 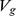 gets damped quickly and efficiency 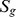 is close to 0.3. The inventory is maintained despite population turnover (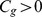) although new inventions may arise and in rare cases occur where a new invention overtakes existing markers. ii) The average preference scores for all invented markers in the memories of all agents for a single experiment. There is one marker with the highest score and two others with lower scores. When a new agent comes in, the average scores go down (see circles) but move back up as the new agent acquires the existing preferences.

This establishes a positive feedback loop between usage and marker preference, which can be expected to lead to a shared minimal marker system. Figure 4 confirms that this kind of self-organization indeed occurs. We see that very quickly (after about 300 games which is 60 per agent) an optimal shared marker system has emerged. This is remarkable because there is no central control nor any prior specification of which or how many markers agents should be using. Initial variation, caused by the fact that any agent has the right to invent new markers when needed, gets damped quickly. The resulting process is similar to self-organizing processes found in natural systems, such as path formation in ant societies, in which large-scale structure arise from local interactions through random behavior influenced by positive feedback loops [32].

It is also remarkable (but obviously a requirement of an adequate model for the evolution of some feature of language) that exactly the same strategy supports cultural transmission. The experiments shown in Figure 4 include population change. Agents get replaced with a probability 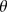, taking with them their existing know how of the prevalent agreement system, and new agents, which are only endowed with the shared vocabulary and the marker strategy but without any marker inventory, are added so that the population remains constant. In the experiments 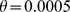 and the population size is 10, which implies that after every game there is a chance of 5/1000 that one of the 10 agents gets replaced. In Figure 4 half of the population has been replaced after 1000 games, but despite of this change, the marker inventory is not affected as new agents learn the markers that are already in use. They occasionally invent new ones but these get damped due to the lateral inhibition dynamics.

### From Formal to Meaningful Markers

The formal marker strategy is an adequate solution to the problem of combinatorial explosion and ambiguity avoidance because as soon as the speaker uses a marker, the hearer can infer that the words to which this marker are attached are about the same object, even if he has never heard the marker before. This strategy could be used by artificial agents that have to build their own communication system from scratch. It is actually used by some sign languages. Arbitrary areas in space are designated as markers and properties or actions involving the same objects are then signed within those areas [33]. But it is not the strategy generally adopted by human languages, which prefer to use meaningful rather than formal markers. For example, the Swahili marker *ki-* is used for the class of inanimate objects, which contains artefacts like baskets. The Latin marker *-arum* expresses plural, feminine and genitive.

It is an interesting question why human languages prefer meaningful markers, given that a formal marker strategy is cognitively simple and highly effective. There is probably not a single explanation. First of all, human memory is able to retain items much more easily when they are meaningful rather than purely formal [34] and they therefore should propagate faster in human populations. More importantly, meaningful markers make it possible to express more meaning with fewer linguistic forms, an important economizing principle of language. If a word already expresses the meaning of a marker, the marker is no longer needed and can be left out. For example, “fille” (Fr. girl) already expresses feminine and therefore does not need an extra marker of gender. Moreover, a marker can introduce additional meanings on top of the meaning suplied by words. This is the case with the Latin “-arum”, which not only carries out the agreement function but also conveys that the referent is plural and genitive.

By what strategy can meaningful markers arise, given that we must assume that there is no external designer and no central control agency that can decide how everybody should speak? We argue that this is by a process similar to the one explored in earlier agent-based models on how a set of shared categories can arise in co-evolution with an emergent system of signs (words, morphemes or other forms) [31], [35], [36]. Each agent requires a concept formation process to generate or select possible features, a symbolization process to generate markers for these features, and a strong coupling between the two.

**Figure 5:**
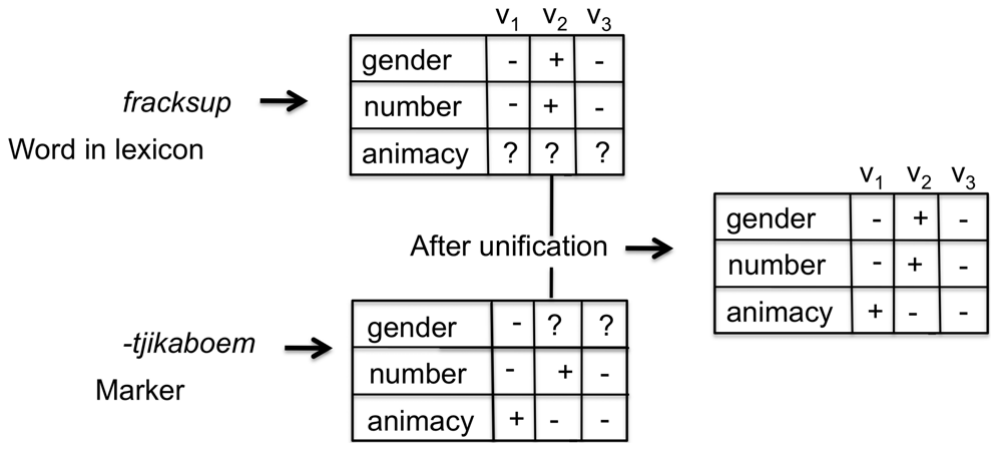
Feature matrices in agreement systems. A feature matrix has rows for the different attributes (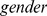, 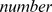, 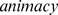) and columns for the possible values of these attributes (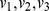) which could correspond for example to 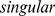, 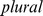 and 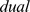 for the attribute 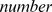. The cells contain + when the relevant attribute value pair is true, - when it is not, and ? when it is open. Two feature matrices are compared using the standard logical unification operator to see whether they fit. Open values may thus be determined. For example, the open values for 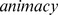 in the feature matrix of *fracksup* become constrained by those specified for the marker *-tjikaboem* and conversely the gender values of the marker become constrained by the word.

**Figure 6:**
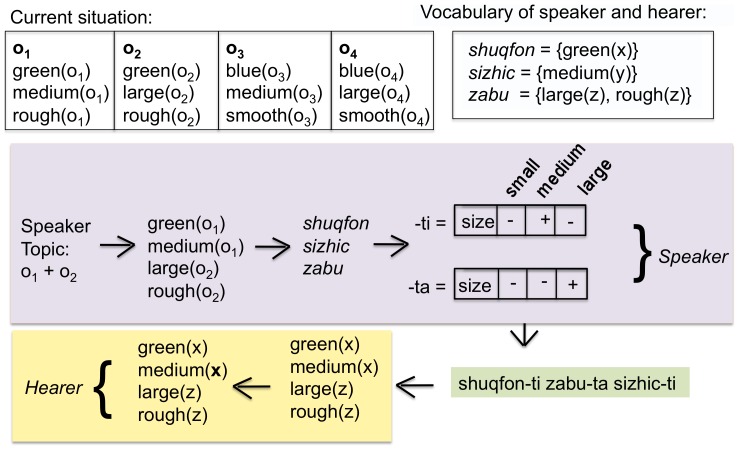
Meaningful marker strategy. When the speaker invents new markers he selects an attribute which is distinctive for the different objects in the utterance (in this case this is 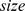) and creates new markers for each value (in this case “-ti” and “-ta” for the two values 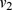 (medium) and 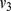 (large) respectively). The hearer is able to guess the semantic features of these markers by using again the principle of distinctiveness.

The first step in devising a computational model for testing this hypothesis is to select an appropriate representation for the semantic features associated with markers, that is adequate for handling the phenomena found in human languages, even the most complex ones. We have used feature matrices, familiar from many areas of linguistics, particularly phonology, and operationalized by computational linguists for agreement systems[37], [38] (see Figure 5 and methods section). Both words and agreement markers have associated feature matrices. The matrix of words is derived from its meaning and the matrix of agreement markers are filled in and aligned by the agents using the following strategy (see Figure 6):

Speaker and hearer keep an inventory 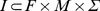 of associations, between a feature matrix 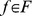, a marker 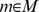 and a score 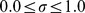.

- When deciding which marker to use for a word 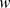 referring to an object 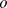, the speaker first searches his existing marker inventory to find a marker that is not yet used earlier in the same utterance and whose feature matrix semantically fits with the feature matrix of 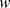. The marker should also have counterparts whose feature matrices fit with the feature matrices of the words used to describe other objects in the utterance and which use the same attributes but have other values. For example, markers using the number attribute are appropriate when each object in the utterance has a different value for number.
  (ii) When no marker can be found, the speaker tries to find an attribute (or more than one) which is distinctive for words used to refer to the different objects in the utterance, creates new markers for each distinctive attribute-value pair, and adds the relevant constructions to his marker inventory 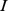. For example, suppose the number attribute is distinctive because one object is singular and the other plural, then two new markers would be invented, for example *-ti* and *-ta*, and feature matrices built with a row for number and positive values in the slots for singular and plural respectively.
- When the hearer encounters unknown markers, he treats them as formal markers and applies them to arrive at a unique interpretation of the utterance. To induce the possible feature matrix of the unknown markers he then follows a strategy similar to the one used by the speaker and creates new constructions for each marker with the relevant feature matrix.

The use of distinctive properties to build the feature matrix of a marker (as opposed to any kind of property) reduces the possible interpretations of an unknown marker for the hearer and thus speeds up learning. But still, there is a risk because there may be more than one distinctive feature combination fitting with a particular context, so that the hearer may induce a different meaning for an unknown marker compared to the meaning assumed by the speaker. Hence, not only variation in the form or meaning of markers but also synonymy (different markers for the same feature matrix) and ambiguity (different feature matrices for the same marker) become unavoidable.

These issues can be dealt with by using again a lateral inhibition dynamics [23], [29]: Each association between a feature matrix 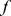 and its marker 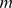 has an association score 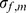, initialized to 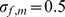 for new associations, and the association with the highest score is preferred by the speaker in production and by the hearer in interpretation. A random choice is made when scores are equally high.

Only the hearer changes the score after a game. In the case of a successful game, the score 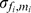 of the used association is increased and its competitors are decreased according to the equations (5-7) with the alignment rate 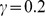:
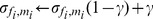
(4)

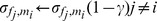
(5)

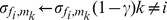
(6)


A competitor is another feature matrix 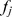 stored in the hearer's memory for the used marker 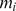 or another marker 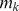 for the feature matrix 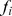. When the speaker is using markers whose features are not distinctive in the current context for the hearer, the hearer formulates alternative constructions based on the current situation and adds them to his inventory. These alternatives compete from now on with the original construction through the lateral inhibition dynamics.

**Figure 7:**
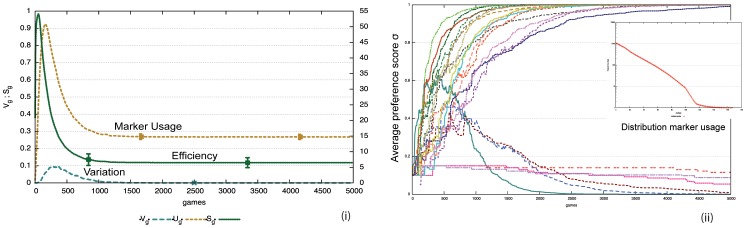
Performance of the meaningful marker strategy. Averages over 50 game series with 10 agents for 5000 games (average of 1000 per agent). There is no population change. i) Agents end up with an inventory 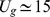 markers. Compared to the formal marker strategy there is more variation which gets damped more slowly and there is considerably less efficiency (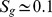). ii) The evolution of marker preferences for a single experiment shows that many markers (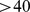) get invented due to the many possible meanings that can potentially be used, but that a subset becomes dominant. The insert displays the frequency of marker usage which shows a long tail Zipfian distribution.

Figure 7 i) shows that this strategy remarkably leads to a self-organizing process whereby agents not only progressively share which markers they prefer (as in the formal marker strategy) but also which feature matrices they should use for those markers. We can see that there is convergence because the variation 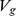 approaches zero and 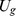, the number of markers being used, reaches a stable plateau around 15 markers. The convergence is reached in a purely bottom-up fashion and in different experimental runs other marker systems may arise. Figure 7 ii) shows the evolution of the marker preferences for a single agent. Compared to the formal marker strategy, there are now more markers because a marker can only apply in specific semantic circumstances. The meaningful marker strategy is also less efficient compared to the formal marker strategy because agents have to coordinate not only which markers they are going to use but also what meaning is attached to each marker. But this disadvantage is outweighed by the fact that markers can contribute new meaning by themselves. The insert in Figure 7 ii) displays the frequency of marker usage which is showing a Zipfian long tail and power law distribution. Markers that express a single feature have a selective advantage because they can apply in more situations and hence their usage dominates.

### Reusing existing words

Given these foundations, we can now operationalize the strategy that is historically found in human languages, namely to derive agreement markers from existing words, such as pronouns or classifiers[14], [15]. It is easy to see why humans would prefer such a strategy. The meaningful marker strategy is not optimal because there are many possible features that could in principle be used as the basis of a marker. Even though agents use distinctive properties for new inventions, there is occasionally still more than one possibility left, so that hearers have to make uncertain guesses about the meaning of unknown markers which have to be filtered out in the collective dynamics. This takes time and scales with the size of the population and the size of the set of possible features, similar to the way emergent vocabularies scale [25]. On the other hand, when agents use existing words as the basis of a new marker, the meaning of the marker is immediately clear to the hearer. It is like pasting on food items in the refrigerator of a student house a label containing a picture of the owner or his or her name, rather than a colorful sticker which does not reflect at all who is the owner.

**Figure 8:**
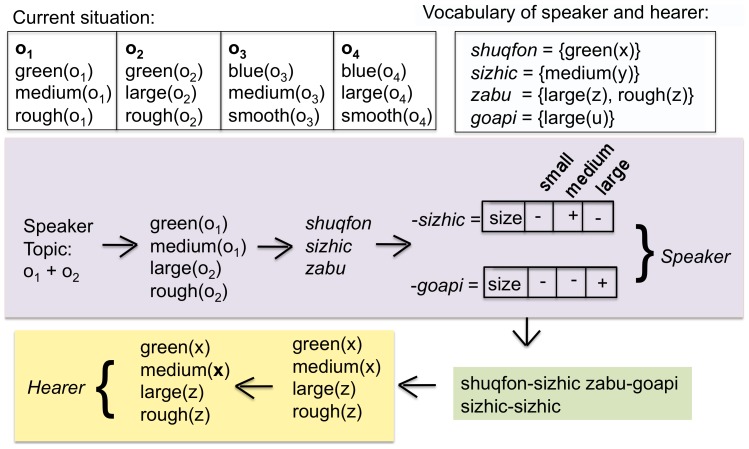
Reuse strategy. Instead of inventing new markers, speakers use existing words which have already the meaning that the marker should express. In this case, the words “sizhic” and “goapi” can be used to express the two feature values for 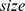. The hearer is now able to infer with more certainty the semantic features for these markers.

Surprisingly, the reuse of existing words for markers can be modeled by a very small change in the meaningful marker strategy discussed earlier, namely by changing the invention step of the speaker (Figure 8). Rather than generating a random string when a new marker is needed, the speaker takes an existing word, which already expresses one feature or a combination of features that distinguishes the topic from the other objects described in the utterance, and uses that as a marker. The speaker prefers the word with the least properties although there is often still more than one possible choice so that the lateral inhibition dynamics that was used with the previous meaningful marker strategy is still needed (see equations 5-7).

**Figure 9:**
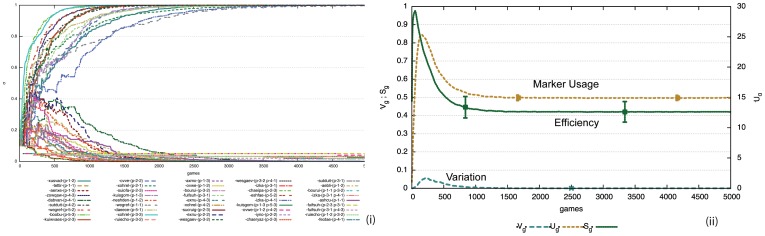
Performance of meaningful marker strategies with reuse. Averages over 50 game series with 10 agents for 5000 games (average of 1000 per agent). i) Agents reach an inventory of a similar size as without reuse (Figure 7) but they reach it in a more efficient way (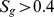). There is less variation that gets damped more quickly. ii) The evolution of marker preferences (single experiment) illustrates that fewer markers are considered and the shared subset becomes dominant more quickly compared to the no-reuse strategy. Below the graph is the list of markers and the properties they express.

**Figure 10:**
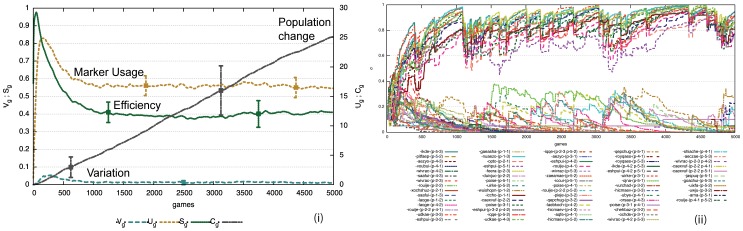
Effect of population change for reuse strategy. i) 50 game series for 5000 games (1000 per agents). Average values are shown with standard deviation. We see clearly that despite population turnover (increasing 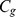), the marker system is transmitted in a stable way once it has emerged. Variation 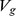 and the size of the marker inventory 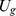 stay at the same level. Efficiency is higher compared to a reuse strategy without population turn over (Figure 9) because in a changing population, markers that were invented early on do not survive. ii) Evolution of marker preferences for a single experiment. The marker preferences are averages for all agents. Coherence dips every time a new agent enters before recovering.

**Figure 11:**
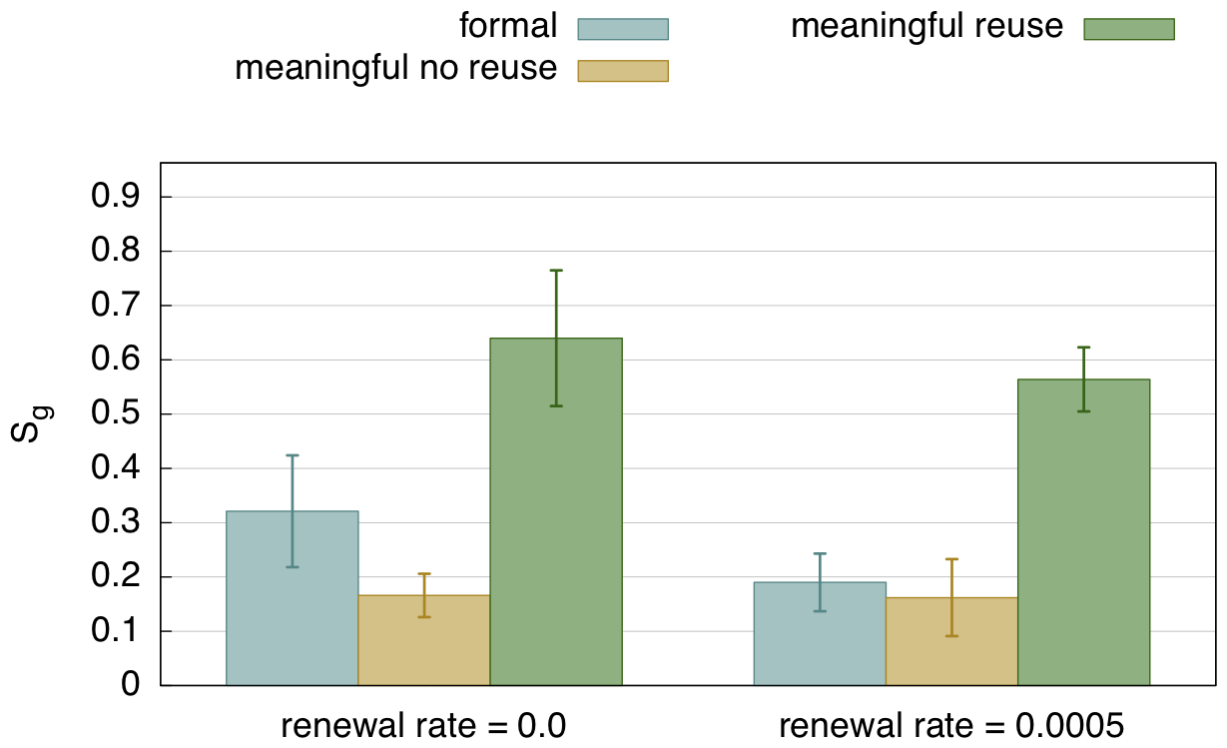
Comparison of strategies. This graph compares the efficiency of the formal marker strategy, the meaningful strategy without reuse, and the reuse strategy for the last 10 games of a run with 5000 games. Left is without population change and right with population change. Average for 50 runs with standard deviation. The reuse strategy is significantly more efficient, even in the case of population turnover. Unsurprisingly, a changing population (shown on the right) makes it harder for the agents to bootstrap an initial system.

Figure 9 i) shows that an agreement system indeed self-organizes again. For the same experimental conditions as in Figure 7 i), agents consider 50% fewer markers and there is fewer variation, which gets damped more quickly. Figure 9 ii) shows the evolution of the preference scores for the total population. We see that a limited set of markers quickly dominates. Figure 10 i) shows that the reuse strategy also works for cultural transmission in the face of population change. Once a system has come off the ground it is stable, even if a new agent occasionally introduces a new invention that then propagates to some extent in the rest of the population. Figure 10 ii) shows the development of the marker preferences. There is new variation introduced by agents coming freshly in the population but it does not challenge the system in place. It is remarkable that this is possible without central control nor prior specification about which (or even how many) markers agents should use. The model shows once again the power of lateral inhibition for explaining how a shared set of conventions can arise in a population. Computer simulations furthermore show that agents can now build a system of shared markers much more efficiently (Figure 11), explaining why this strategy is universally preferred in the initial stages of agreement marking in human languages.

### Phonological Reduction

**Figure 12:**
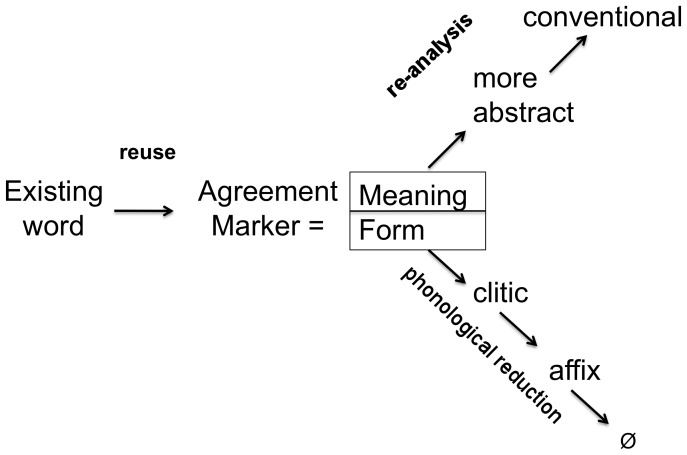
Grammaticalization. Human natural language systems historically build agreement systems by reusing existing words. The form of these words then undergoes erosion by phonological reduction processes until the marker may get lost entirely. Their meaning becomes more abstract and semantic features become purely conventional rather than grounded in the meaning of the word, through coercion.

We now turn to models for the grammaticalization processes universally observed in human languages (Figure 12). First, how can we explain that agreement markers erode, even up to a point where this leads to syncretism (which means that the same marker has more than one possible function) and potentially to a destabilization of the whole system? We appeal again to economizing principles. Human speakers tend to minimize the amount of sounds in an utterance by eliminating consonants or vowels from word forms so that speakers need less articulatory effort and hearers require less auditory processing. This most definitely has happened with agreement markers. For example, the old High German singular masculine or neutral dative article *dëmu* has eroded to become *dem* in contemporary German [39]. This kind of phonological erosion of a marker has the additional advantage that markers become distinct from the word that was initially used for the marker, which makes parsing easier because multiple uses of the same form are eliminated. On the other hand, erosion may lead to syncretism. For example, the old High German singular feminine dative or genitive article *dëru* and the plural genitive *dëro* have both eroded to *der*, thus creating a confusion with each other and with the existing article *der* for singular masculine nominative. Erosion also eliminates the advantage of the reuse strategy, because the hearer has no clues anymore about the possible meaning of an unknown marker. This example shows that language must balance multiple constraints which occasionally are in conflict each other. There is no optimal solution and this explains why languages keep evolving, moving around in the landscape of possible languages, sometimes optimizing one factor (for example articulatory efficiency) at the cost of another one (speed up learning).

We have studied the effect of phonological reduction by adding a tendency for optimizing articulation on top of the reuse strategy. Sophisticated models of speech articulation exist and they predict the kind of errors and variations that speakers tend to introduce and how they influence sound change [40]. We have used a simpler model that nevertheless brings out the language dynamics clearly. Speakers optimize articulation by leaving out the last consonant or vowel of a marker with a certain probability 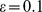. Hearers are flexible enough in their parsing of markers to recognize that a truncated form is a variant of an existing marker, as long as it deviates for only one consonant or vowel. This maintains an adequate level of communicative success and does not diminish the effectiveness of markers to cut down combinatorial complexity and semantic ambiguity. But how can we explain that a variant might itself become the norm and in turn become the subject of further optimizations?

**Figure 13:**
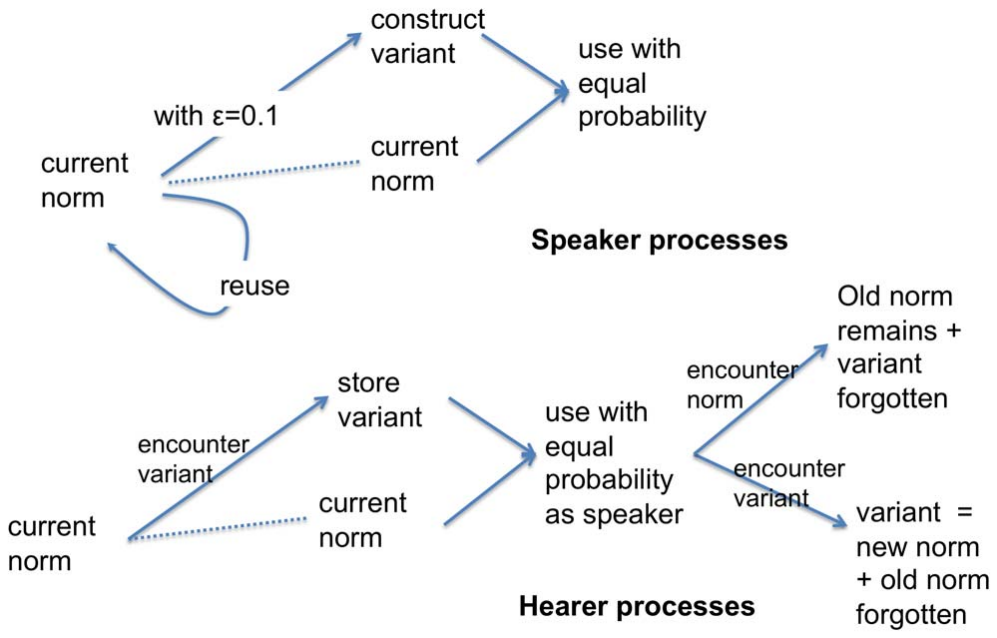
Phonological reduction strategy. Speakers construct a new variant by phonologically reducing a marker's form with a probability 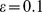. The hearer is able to recognize a variant if it deviates only for one consonant or vowel and will adopt it as the new norm for the marker when the variant is encountered twice.

It turns out that the lateral inhibition learning rule (equations 5-7) is not adequate to achieve this language dynamics because a variant produced by accidental or deliberate phonological reduction does not have enough of a chance to overturn the system that is already in place. Instead we will use a more radical inhibition dynamics [28] inspired by voter models as developed in game-based social science research (see Figure 13). When an agent produces or encounters a truncated marker, he stores it in his inventory as a new variant of the original marker, and later uses the original or the truncated form with equal chance. However, as soon as an agent encounters the truncated marker for a second time, he adopts it as the new norm and the old form is discarded. It is possible that the agent encounters again the previous older form which may then be re-adopted and reused if it is encountered more than once. However, at some point, there are enough agents using the new variant so that the whole population shifts in a phase transition. When a form has eroded to a point where a confusion may arise with another marker on further reduction, the agent sticks to the existing norm, because this would otherwise lead to syncretism (same form different feature matrices) and possibly a partial destruction of the agreement system. Syncretism and destabilization does happen in human languages, as attested in the evolution of the German case system [41] or the disappearance of the English case system [7], but we do not want to address this phenomenon in the current experiments (see [42] for preliminary agent-based models in this direction).

**Figure 14:**
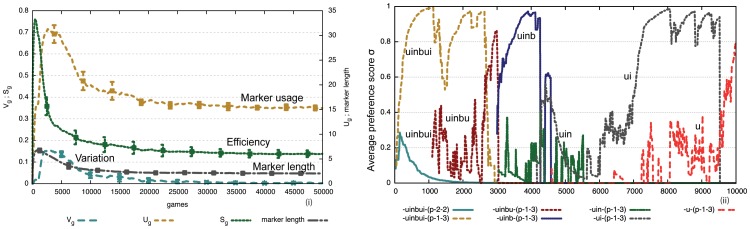
Performance of the phonological reduction strategy. i) Average performance values for 50 game series for a total of 50,000 games (average of 10,000 per agent). The probability that the speaker phonologically reduces a marker is 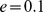 per game. We see that the length of the markers progressively diminishes, thus reducing articulatory effort, but variation 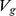 does not increase, implying that the system is stable. The marker inventory size remains constant as well. ii) Trace of the changes to a marker in a single experiment. The marker *-uinbui* erodes progressively to *-u*. A truncated variant is typically present for a while in the population until a phase transition happens and it becomes dominant.

Figure 14 shows the outcome of a computer simulation of this phonological reduction strategy. An agreement system based on meaningful markers is emerging using the meaningful marker strategy. But after agents reach a stable level of performance (in the experiment this is typically after 200 games per agent), they occasionally introduce phonological reductions with probability 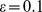 and this leads to the erosion of the original markers. Figure 14 i) shows that the average marker length is decreasing from an average of 7 to 4 consonants and vowels, without affecting performance. There is greater variation 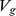 in the population because there are always different variants of the same marker in use, but this generally does not have an impact because agents are able to recognize them as a variant of their own norm.

Figure 14 ii) shows a typical example how a marker (in this case “-uinbui:”) erodes. At the beginning there is still competition for two meanings for this marker (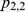 and 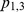) but soon the second meaning dominates. The form gets phonologically reduced in the sequence “-uinbui” 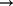 “-uinbu” 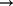 “-uinb” 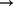 “-uin” 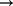 “-ui” 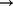 “-u”. At this point reduction stops because speakers are able to detect that otherwise the function of the marker would get lost.

These results are significant because they show, for the first time, how phonological reduction carried out by individual agents can lead to marker erosion, without destroying the functioning of the agreement system as a whole and even though there is no central controlling agency ensuring that shared norms are maintained.

### Coercion

Historical linguists have also observed that agreement features generalize so that markers can be applied in situations which do not fit with the original meaning and so the features become purely conventional (Figure 12). Why does this happen? When semantic features that are grounded in the real world (like animacy, number or sex) are chosen as dimensions for a marker's feature matrix, situations unavoidably come up where these features cannot apply. Of course a new marker could then be introduced, possibly based on another existing word. Indeed this is what happens in the strategies reported earlier. But unavoidably, language users then end up with more markers than is strictly necessary. Some languages do have a large set (for example, the North-West Amazonian language Tariana has no less than 100 markers for subject verb agreement [43]) but a large inventory makes it harder to acquire the language and requires more cognitive effort for storing and retrieving markers.

One way to solve this problem is to make the markers semantically more general. This process is called semantic bleaching and is indeed observed systematically in the historical evolution of agreement markers [44]. Another way is to ‘re-categorize’ a word by assigning agreement features to it in the vocabulary, thus coercing this word so that the features of a marker fit, even though the objects referred to by this word do not have these features from a purely semantic point of view. For example, a word describing an inanimate object like *table* has been categorized to be of feminine gender in French (*la table*), even though a sex distinction does not make sense for tables. Natural languages clearly use both strategies in parallel, but we have operationalized here only a coercion strategy and tested it in computational agent-based simulations.

To start modeling the coercion strategy we must introduce two extensions of the model.

1. The literature on agreement makes a distinction between controllers and targets [2], and only words belonging to certain syntactic categories can be controllers. A controller determines the agreement features of its targets. For example, nouns are controllers of articles and adjectives in the internal agreement within noun phrases. Thus, you can say in French “une table est un objet” (a table (feminine) is an object (masculine)), illustrating that a different gender can be used for the same object depending on the noun chosen as the locus of reference. A word group has obligatory a controller and when the agent vocabularies are initialized, some words are randomly assigned the status of controller.

2. We have already seen (Figure 5) that words can have open cells for features that are not grounded in the meaning of the word. For example, *fille* (Fr. girl) has the semantic feature feminine because the person referred to by this word is semantically of female sex. But other words, such as *chaise* (Fr. chair) do not have any natural semantic grounding of gender and this semantic feature is therefore undecided and hence potentially open to a conventionalization process.

**Figure 15:**
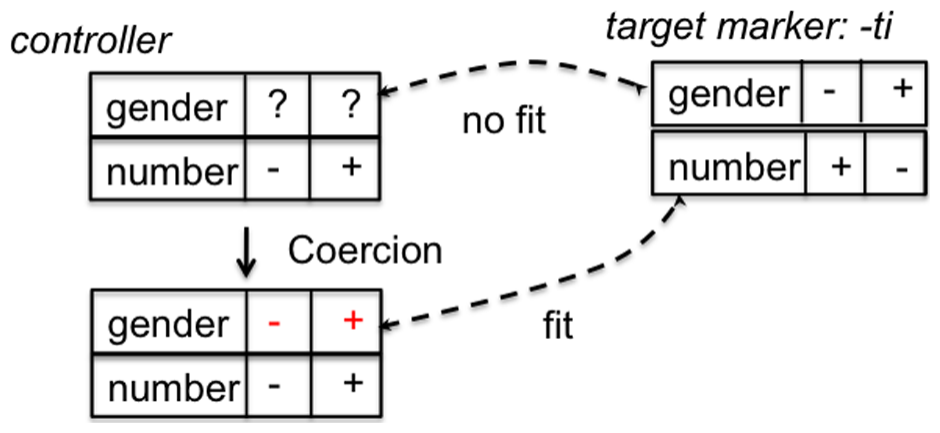
Coercion. Suppose that the speaker wants to use the marker *-ti* but that the relevant word has no values for gender. The feature matrix of the word can then be ‘coerced’ by assigning it the values of the feature matrix of *-ti* so that now there is a semantic fit.

We hypothesize that conventionalized markers originate when agents use another invention step in the meaningful marker strategy. Recall that when the speaker could not apply an existing marker because there are no markers that match with the semantic features of the words in an utterance, he looks for a combination of distinctive properties and creates a new marker (or reuses an existing word) with this combination as its meaning. This step is still needed to build an initial set of markers, but now we add an extra step: when there is already a marker which partially matches with the topic and the controller used to introduce the topic is undecided with respect to the other features of that marker, the agent can assign by convention the values of the marker to the controller and thus coerce it to be compatible with the marker (see Figure 15). When this coercion is successful it is stored and gets into competition with the earlier construction for the controller.

As with earlier strategies, there is the possibility that agents diverge on which controllers to coerce and how, so that a mechanism is needed to ensure that the population reaches convergence on marker usage. We have used again a lateral inhibition strategy. When the hearer detects that a marker is applied to a controller which has an incompatible feature matrix, he constructs a new feature matrix for the controller and stores that as a variant in his memory. From then on this feature matrix competes with the others associated with the same controller. After a successful game the used association is increased by the hearer and its competitors decreased according to equations (5-7).

**Figure 16:**
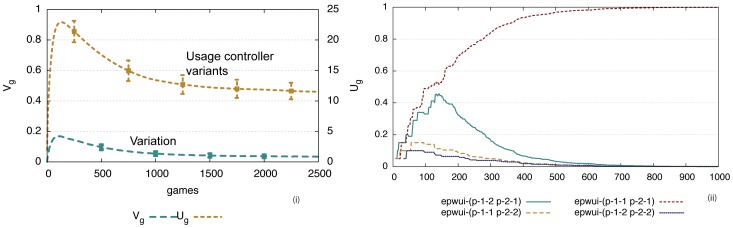
Performance of coercion strategy. i) Performance summary for 50 game series of 1000 games (200 per agent), average values with standard deviation. We see that variation, which is unavoidable due to the different possible ways in which re-categorization can be deployed, gets damped quickly. The number of markers is reduced compared to the plain reuse strategy (figure 9 i). ii) Development of which feature matrix becomes associated with the controller *epwui*. This controller started out with an empty feature matrix, i.e. no semantically grounded features. There are different ways in which it can be re-categorized with respect to the existing marker inventory. In this experimental run, a feature matrix with a positive value for 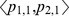 has become dominant.

**Figure 17:**
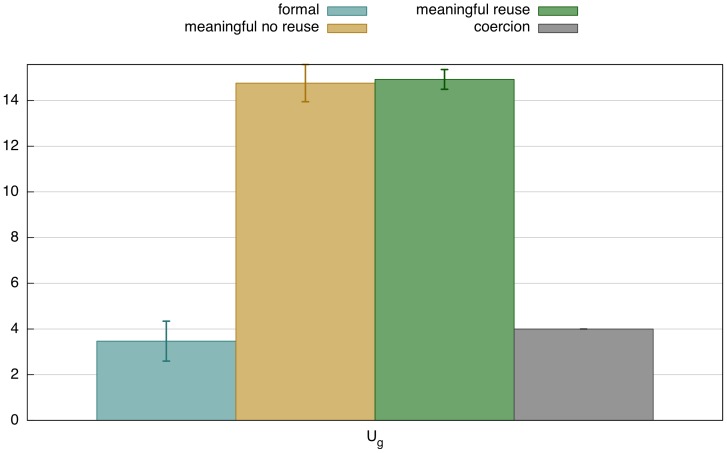
Comparison of inventory size. This graph compares the main strategies presented here with respect to the size of the marker inventory. It shows that meaningful markers require a larger marker inventory (because they have semantic constrains). However, using a coercion strategy, agents manage to function with a similar inventory size for meaningful markers as for formal markers, thus significantly reducing effort for learning and memory.

Figure 16 shows the results of computational experiments with this coercion strategy. The vocabulary is initialized in such a way that some subset of words can function as controllers and the rest as targets. The set of controllers is further split into a subset where all semantic features are grounded in the lexical meaning, and a set which is open for some attributes of the feature matrix. For example, a controller may have a concrete value for number but not yet for gender. Agents are also initialized with a minimal randomly generated marker system. Then the evolutionary process starts. Figure 16 i) shows that a functioning agreement system develops further and becomes shared in the population. The main advantage of the coercion strategy is that 50% fewer markers are in the agent inventories because the feature matrices of controllers adjust to the available markers (Figure 17). Figure 16 ii) shows how the lateral inhibition dynamics causes one of the competing feature matrices of a particular controller (“epwui”) to become dominant.

These experiments are the first agent-based simulations showing how coercion through re-categorization can allow agents to minimize their marker inventory, as part of on-going communications and without destroying their existing system. On the contrary, by re-categorizing existing markers they become used more broadly and lead to a more efficient smaller marker inventory. It is of course possible to make the coercion process much more complex, for example, by modeling common sense knowledge and inference, or by operationalizing analogy; strategies that are clearly used for coercion by human language users. However we kept the model deliberately simple because its main purpose is to understand through a minimal model what language dynamics coercion generates and thus to study the cognitive and cultural mechanisms underlying the grammaticalization processes universally found in human languages.

## Conclusions

We presented here the first agent-based models to explore how and why a grammatical agreement system may originate and get culturally transmitted in a process of cultural invention and social learning, based on the hypothesis that agreement systems are useful to avoid combinatorial explosions in parsing and semantic ambiguity in interpretation. Agreement systems thus help to minimize cognitive effort and maximize communicative success. After demonstrating how formal markers could arise, we presented strategies showing how meaningful markers could originate, and how markers could become recruited from existing words. We demonstrated also how recruited words could erode to lead to greater articulatory efficiency, at a cost of giving fewer hints for new language users, and how coercion helps to apply an agreement system more broadly so that fewer agreement markers are needed. These various steps and their effect are summarized in Table 1.

**Table 1:** Summary.

Strategy	Description	Advantage	Drawback
Formal Marker	Adds arbitrary marker to co-referential words	Avoids combinatorial search in parsing and semantic ambiguity	Only motivation for a marker is its frequency
Meaningful Marker	A marker now carries meaning	Allows more meaning to be expressed with the same number of forms.	Meaning of marker not transparent to learner
Reuse	A marker is based on reusing an existing word	Learner can immediately guess its meaning	Still many more markers than with formal marker strategy
Phonological Reduction	The form of a marker is shortened	Increased articulatory efficiency	Advantage of reuse strategy is partially lost
Coercion	Semantic features of words are re-categorized so that marker becomes applicable	Reduction of marker inventory	Danger of syncretism

Overview of different strategies with their main advantages and drawbacks. The advantages are cumulative and the drawbacks of one strategy are resolved by the next one.

More generally, the models presented here are a powerful illustration of agent-based modeling, which is a novel way to study the origins of grammar. The grammatical structure of language is seen as originating from the need to handle issues that unavoidably come up when building a symbolic communication system: combatting combinatorial explosions, avoiding semantic ambiguity, improving learnability, decreasing articulatory effort. Agent-based simulations help us to expose these issues in very precise ways and test whether certain strategies can handle them. Agent-based experiments are complementary to other approaches that also investigate how economizing principles shape human language: by conducting artificial language learning experiments [45], or by observing language games with human subjects [46], [47]. Most areas of grammar remain to be explored but the agent-based models presented here already clearly show the power and promise of this methodology.

## Materials and Methods

The following measures are being used to show the results of the different experiments:

(i) The *usage* 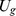 is the number of different markers effectively used by the population in the interval 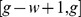, where 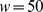 is the size of a sliding window over a consecutive series of games. When 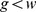 we measure the number of different markers for the interval 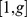.

(ii) The *variation* 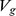 measures in how far agents have a shared usage inventory within the interval 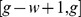. It is computed with the following equation:
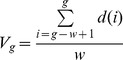
(7)

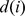
 compares for a game 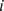 the marker used by the speaker with what the hearer would have used for the features selected by the speaker. If they are equal 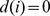, otherwise 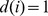.

(iii) Let the *inventory size* 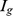 be the total number of distinct markers invented by the whole population up till game 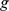, then the *effective usage* 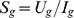 captures how well superfluous inventions could be avoided within this interval. This gives an indication of the *efficiency* of the strategy.

(iv) 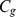, the population change at game 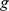, is measured as the number of new agents that have entered and been removed from the initial population, from an initial state which is 10 agents.

In all experiments, the ontology, situation model and vocabularies are abstract. The situations in the world are generated on the basis of an ontology 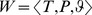. 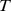 is a set of possible types. 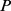 is a set of possible properties. A property 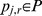 is an attribute-value pair with attribute 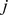 and value 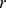. Each attribute 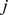 has a limited number of possible values. For example, an attribute could be animacy and possible values living, and non-living. We typically use 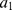,..., 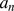 as names of attributes and 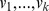 as names of values. For example, 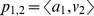 is the name of a property with attribute 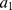 and value 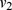. 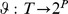 defines for every type 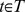 the subset of 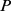 that constitutes the possible properties of 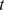. For example, there could be a type human with possible properties for the attributes animacy, sex, and size. In all experiments reported here, there are 5 attributes with 3 possible values each, and 10 possible types which each have between 1 and 5 randomly chosen attributes and between 1 and 3 possible values for these attributes.

A situation model 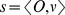 contains a set of objects 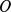 and a valuation function 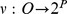, which defines for every object 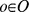 the properties that are true for 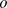 in 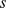. Every object 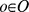 is an instance of a type 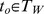 and 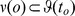. To assert that 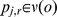 we also write 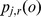. When it is unknown to which object a property applies, we write 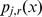, where 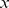 is a variable. The situation model is shared by both participants in a language game. In the experiments reported here, there are 3 objects in each situation model, out of which a set with 1, 2 or 3 members is chosen as the topic with equal probability. Agents are initialized with a shared pre-defined vocabulary 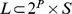 which consists of associations between a set of properties and a word, from the set of possible words 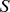.

The strings used for words are generated randomly from a finite set of characters. For example, *pishou* could mean 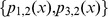. The vocabulary is constructed on the basis of the types 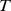 defined in the ontology. To focus on the issue of agreement, the vocabulary does not contain synonyms (same meaning, multiple words) nor any polysemy or word ambiguity (same word, multiple meanings), so that the complexity of interpretation is solely dependent on the fact that words are being combined rather than on there being multiple meanings or multiple forms of the words themselves. On the other hand, words may have overlapping meanings. For example, there could be a word *zwampr* for 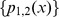 in addition to *pishou* which covers 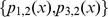. Given the experimental parameters adopted in the simulations, there is 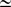 25% chance that 1, 2, or 3 word utterances are produced but there are also 4-word (15%) and 5-word (5%) utterances.

The vocabulary construction process guarantees that for each type 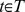, there are enough words to cover all the properties in 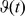. Concretely, all possible meaning combinations with a maximum of 3 attributes are generated for each type and a word is constructed for every combination in this set. To guarantee that there are no synonyms, only one word is constructed for each combination of properties even if it is useful for more than one type. For the experimental parameters used here, this leads on average to an agent vocabulary of 270 words with the majority (75%) covering three attributes.

The semantic and syntactic agreement features are represented using feature matrices [37], [38]. A feature matrix contains rows for each attribute and columns for the possible values (Figure 5). A cell contains + if the value is required for the attribute, - if it is not, and a question-mark if it is open what the value can be. Both, words and markers, have an associated feature matrix:

- The feature matrix of a word is built by converting its properties to positive values in the appropriate cells and by using question marks in case the value is open. For example, the German word “Mann” (man) constrains gender to be masculine and number to be singular but is open with respect to case because it can be nominative, dative or accusative. The positive values in the feature matrix of a word are said to be grounded (in the lexical meaning of the word).
- The feature matrix of a marker is entirely conventional and gets progressively filled in by the invention and learning activities of the agents. Some of the cells in the feature matrix of a marker can remain open as well. For example, the German adjective marker “-en” is used with singular masculine adjectives which cannot be nominative or accusative but it is open whether the case is dative or genitive.

In order to determine whether a marker can be used with a particular word, the values in the respective cells are compared in a two-way matching process, using the unification operation, widely used in current language parsers [48]. When a cell has an open value, it takes on the value of the corresponding cell in the other matrix. This models how, for example, the agreement marker “-m” in the German possessive “meinem” in “meinem Bruder” (my brother) constrains “Bruder” to be dative, and how in turn “Bruder” constrains “meinem” to be masculine instead of neuter. When two feature matrices can be unified we say that they fit.

The implementation of the lexicon and the grammar is based on the Fluid Construction Grammar formalism [49]. Details on the implementation of the lexicon and the grammar for each strategy are provided using the following web resource as supporting information: http://ai.vub.ac.be/materials/plos-agreement/. This site allows interactive inspection of constructions, parsing and production processes, and transient structures. A full example is also given of a complete interaction with the formal marker strategy.

## Supporting Information

Link S1 **Support Materials.**
(PDF)
