# Supplementary material for: Agent-Based Models of Strategies for the Emergence and Evolution of Grammatical Agreement
Source: PLoS One. 2013 Mar 18;8(3):e58960. doi: 10.1371/journal.pone.0058960 (PMC3601110; doi:10.1371/journal.pone.0058960)
Supplement: Link S1 — Support Materials. (PDF) [file pone.0058960.s001.pdf]

Please go to the following site for supplementary information:

http://ai.vub.ac.be/materials/plos-agreement/
